# Metastatic renal clear cell carcinoma to the rectum, lungs, ilium, and lymph nodes: A case report

**DOI:** 10.1097/MD.0000000000005720

**Published:** 2017-01-10

**Authors:** Guoyang Zheng, Hanzhong Li, Ji Li, Xuebin Zhang, Yushi Zhang, Xingcheng Wu

**Affiliations:** a Department of Urology; b Department of Pathology, Peking Union Medical College Hospital, Chinese Academy of Medical Sciences and Peking Union Medical College, Beijing, China.

**Keywords:** case report, clear cells, FDG-PET/CT, metastasis, rectum, renal cell carcinoma

## Abstract

**Background:**

Renal cell carcinoma metastasizing to rectum is very rare, and the unusual metastatic sites should be paid attention to during the follow-up of renal cell carcinoma.

**Case summary:**

We describe a case of a 65-year-old male who was diagnosed with metastatic renal cell carcinoma to rectum 10 years after the right radical nephrectomy. Histopathology and immunohistochemical examinations contribute to making differential diagnosis between rectal metastasis of renal cell carcinoma and primary rectal clear cell carcinoma. Positron emission tomography-computed tomography with fluorodeoxyglucose shows hypermetabolic activity in upper rectum and other sites of metastasis at the same time.

**Conclusion:**

Possibility of unusual metastatic sites of renal cell carcinoma such as rectum indeed exists, which should not be ignored in the surveillance after resection of the primary tumor.

## 1 Introduction

Renal cell carcinoma (RCC) is the most common type of renal tumor, accounting for about 2% to 3% of adult malignancy.^[1,2]^ It is reported that approximately 20% to 40% of patients will develop distant metastatic or locally recurring disease after radical nephrectomy.^[3]^ The frequent sites of metastasis are successively the lungs, lymph nodes, bones, liver, adrenal glands, and brain,^[4]^ while it is extremely rare for RCC to metastasize to rectum. Here, we present a case of metastatic RCC to the rectum, bilateral lungs, right ilium, and pulmonary hilar lymph nodes, after initial radical nephrectomy.

## 2 Case report

A 65-year-old male underwent a right radical nephrectomy 10 years ago, and the postoperative pathologic examination revealed renal clear cell carcinoma (RCCC). After operation, he received a course of chemotherapy. From then on, he did not accept any treatment or follow-up examinations related to RCC. In 2008, 2 years after nephrectomy, he received a rectal polyp resection by colonoscopy, and the pathologic examination revealed primary rectal benign polyps. Since then, he received an electronic colonoscopy every 1 to 2 years until the year 2016, when a protruded lesion was found in his rectum.

**Figure 1:**
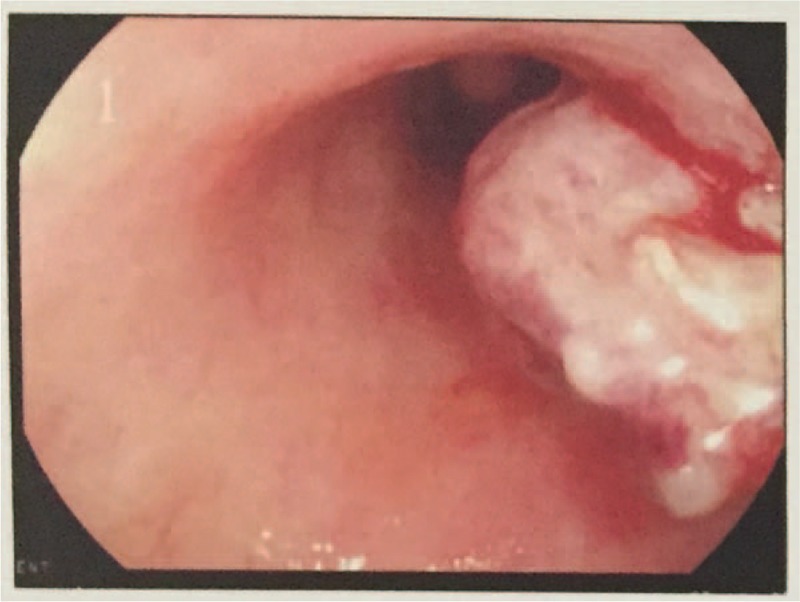
View of colonoscope: a hyperemic and fragile mass without pedicle, covered with some sphacelus, 1.2cm × 1.5 cm size, protruded from rectal mucosa, 10 cm distant from anus.

**Figure 2:**
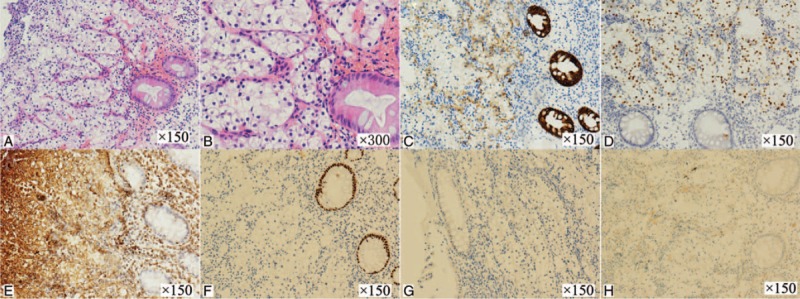
Micrograph of the biopsy specimen and immunohistochemical examination results. (A) HE staining image, ×150. (B) HE staining image, ×300. (C): AE1/AE3 (+), ×150. (D) PAX-8(+), ×150. (E) Vimentin (+), ×150. (F) CDX-2(−), ×150. (G) Melan-A (−), ×150. (H) α-inhibin (−), ×150.

The electronic colonoscope examination showed a hyperemic mass covered with sphacelus, approximately 1.2 cm × 1.5 cm size, which was protruded from rectal mucosa (Fig. 1). The mass was fragile and tended to hemorrhage. Biopsy tissue was confirmed as metastatic RCC to rectum by the result of histopathology and immunohistochemical examination. Under the microscope, there were amounts of large and anomalously shaped clear cells with abundant clear cytoplasm and nuclear atypia consistent with RCCC, forming nests or aciniform structure surrounded with slender small vascular tissues (Fig. 2). Immunohistochemical examination revealed cytokeratin pan (AE1/AE3) (+), paired box (PAX)-8 (+), Vimentin (+), caudal typehomebox transcription factor (CDX)-2 (−), Melan-A (−), and α-inhibin (−) (Fig. 2).

**Figure 3:**
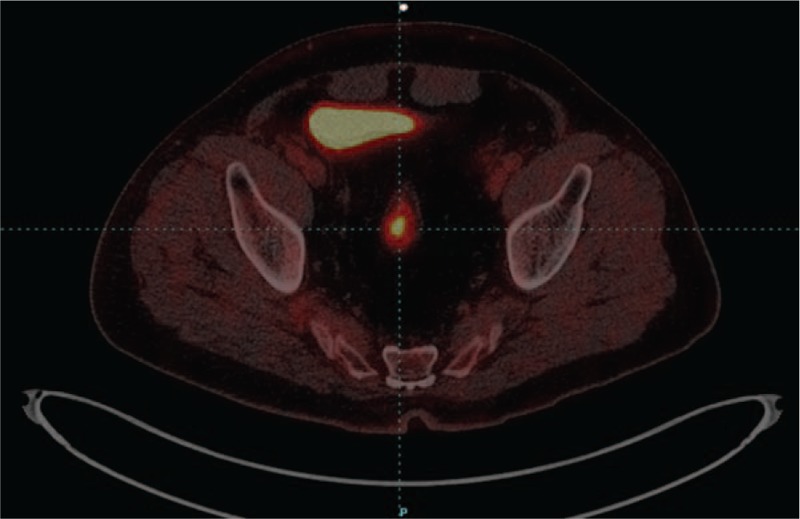
Axial view of FDG-PET/CT: hypermetabolic activity in upper rectum with average SUV of 5.0 and SUVmax of 10.0, 1.6 cm × 2.8 cm × 1.8 cm sizes.

**Figure 4:**
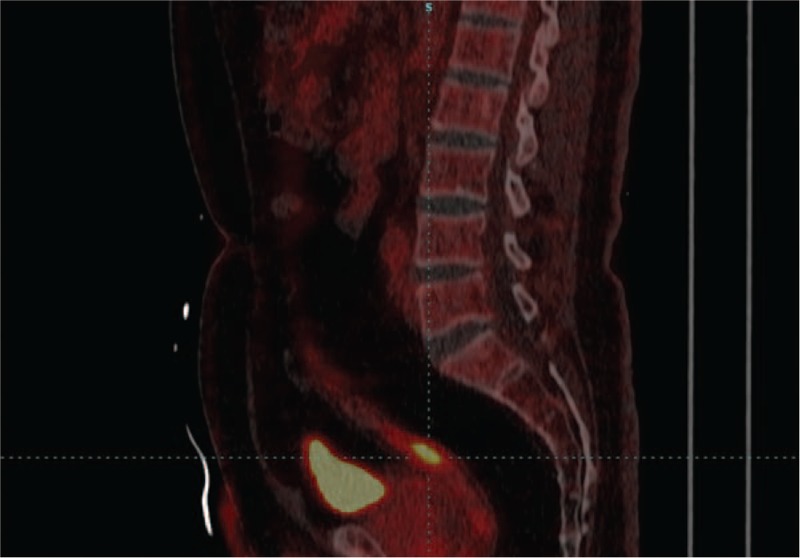
Sagittal view of FDG-PET/CT: abnormal increased radioactive uptake in the upper rectum.

Positron emission tomography-computed tomography with fluorodeoxyglucose (FDG-PET/CT) examination was performed, which showed an abnormal increased radioactive uptake in the upper rectum, 1.6 cm × 2.8 cm × 1.8 cm size, with an average standardized uptake value (SUV) of 5.0 and SUVmax of 10.0, indicating possibility of malignancy (Figs. 3 and 4). In addition, we discovered multiple metastases in bilateral lungs, lymph nodes, and right ilium. In consideration of the existence of multiple metastatic lesions, the patient received targeted therapies by using sunitinib. After 3 months of treatment with sunitinib, the CT images showed that the metastatic lesion of rectum was stable, as well as other metastasis, and the patient is still being followed-up now. The patient is informed consent, and the timeline is listed in Table 1.

**Table 1:**
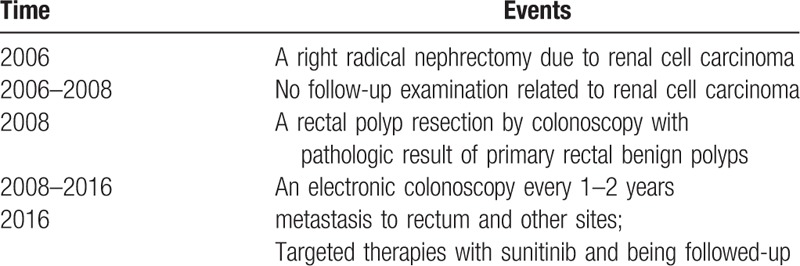
Timeline.

## 3 Discussion

We reported a rare case of metastatic RCC to rectum, furthermore showing the characterization of FDG-PET/CT images of metastatic RCC in rectum for the first time. The definite diagnosis was based on the medical history of RCCC, hypermetabolic activity in FDG-PET/CT images, result of colonoscope, and histopathologic examination with immunohistochemical staining. However, the patient has not experienced symptoms associated with rectal lesions, for instance of hematochezia or changes in bowel habits. There were only 2 cases of metastatic RCC to rectum previously being published. One patient with hematochezia was found to have a submucosal mass with ulcerated area in rectum by colonoscopy and ultrasound, after receiving a left nephrectomy for RCC.^[5]^ The other patient with metastatic RCC to rectum directly received surgery, without any endoscopic or sonographic images.^[6]^

The exact mechanism of metastasis of RCC is still unclear, but the blood supply and thin tissue of rectum may be not appropriate for the development of RCC, compared with common metastatic sites such as lungs. We assume that maybe the high malignancy and strong invasive ability of tumor lead to metastasis to rectum, because the presence of such a rare metastatic site of RCC usually indicates relatively poorer prognosis. Other unusual metastatic sites of RCC include colon, intestine, stomach, pancreas, gallbladder, prostate, thyroid gland, and skeletal muscle.^[7–11]^ These rare metastatic sites were usually accompanied by multiple metastasis in the whole body, and the period from the initial diagnosis of RCC to metastasis may range from several years to more than 10 years.

Although the primary clear cell carcinoma in rectum is rare,^[12]^ it is necessary to make a differential diagnosis between metastatic RCC to rectum and primary rectal clear cell carcinoma. Identification of source of the tumor could influence the establishment of therapeutic strategy. The 2 types of tumors both contain plentiful clear cells, which are typically round or polygonal with abundant clear cytoplasm, while the characterization of histopathology is different. Cells of RCC mainly form nests or aciniform structure in various shapes containing a network of delicate vascular sinusoids interspersed between nests or acini of tumor cells, rarely developing authentic glandular tube. In contrast, cells of primary rectal clear cell carcinoma mostly arrange in glandular tube structure similar with traditional adenoma, partially forming cribriform architecture or solid nest-like structure as well.^[13]^

In addition, the difference in immunohistochemical markers may contribute to differential diagnosis between the 2 types of tumors. RCCC mainly shows positive staining for RCCA, CD10, PAX-8, PAX-2, Vimentin, and AE1/AE3. On the contrary, CK20 and CDX-2 are negative markers for diagnosis of RCC, which were both positively expressed in carcinoma originating from colorectum. This patient has a clinical history of the previous diagnosis of renal cell cancer. Moreover, immunohistochemical examination revealed positive expression of AE1/AE3, PAX-8, and Vimentin, together with negativity for CDX-2. On the basis of above, we could confirm that the mass in upper rectum of the patient was metastasis from RCC.

In this report, FDG-PET/CT images of metastatic RCC in rectum showed hypermetabolic activity in upper rectum with an average SUV of 5.0 and SUVmax of 10.0, which was a relatively high level. Higher SUV usually indicates higher malignancy of tumors, stronger invasiveness of tumor cells, and worse prognosis of patients. Better yet, metastasis of other sites such as bilateral lungs and right ilium could be discovered at the same time, contributing to systematically evaluating the patient's condition and prognosis. FDG-PET/CT is a very effective method to find some distant and infrequent sites of metastasis in surveillance on RCC, due to its good specificity and sensitivity. It could supply anatomical details such as CT as well as functional information of small sized tumors indiscoverable on CT, through a single procedure of body scanning.^[14]^ Therefore, FDG-PET/CT is an optional valuable examination for appropriate case in order to increase the concern about metastatic disease and to influence further evaluation and management,^[15]^ because it could efficiently detect small lesions and identify malignant cases.

## 4 Conclusion

Regular examinations and strict follow-up after nephrectomy are necessary for patients with diagnosis of RCC. Rectum and other unusual metastatic sites of RCC should not be ignored in surveillance on RCC. In addition, FDG-PET/CT contributes to discovering rare metastasis of RCC and other multiple metastatic sites of whole body at the same time.

Ethical review is not necessary, because this is a case report. And the patient is informed consent.
